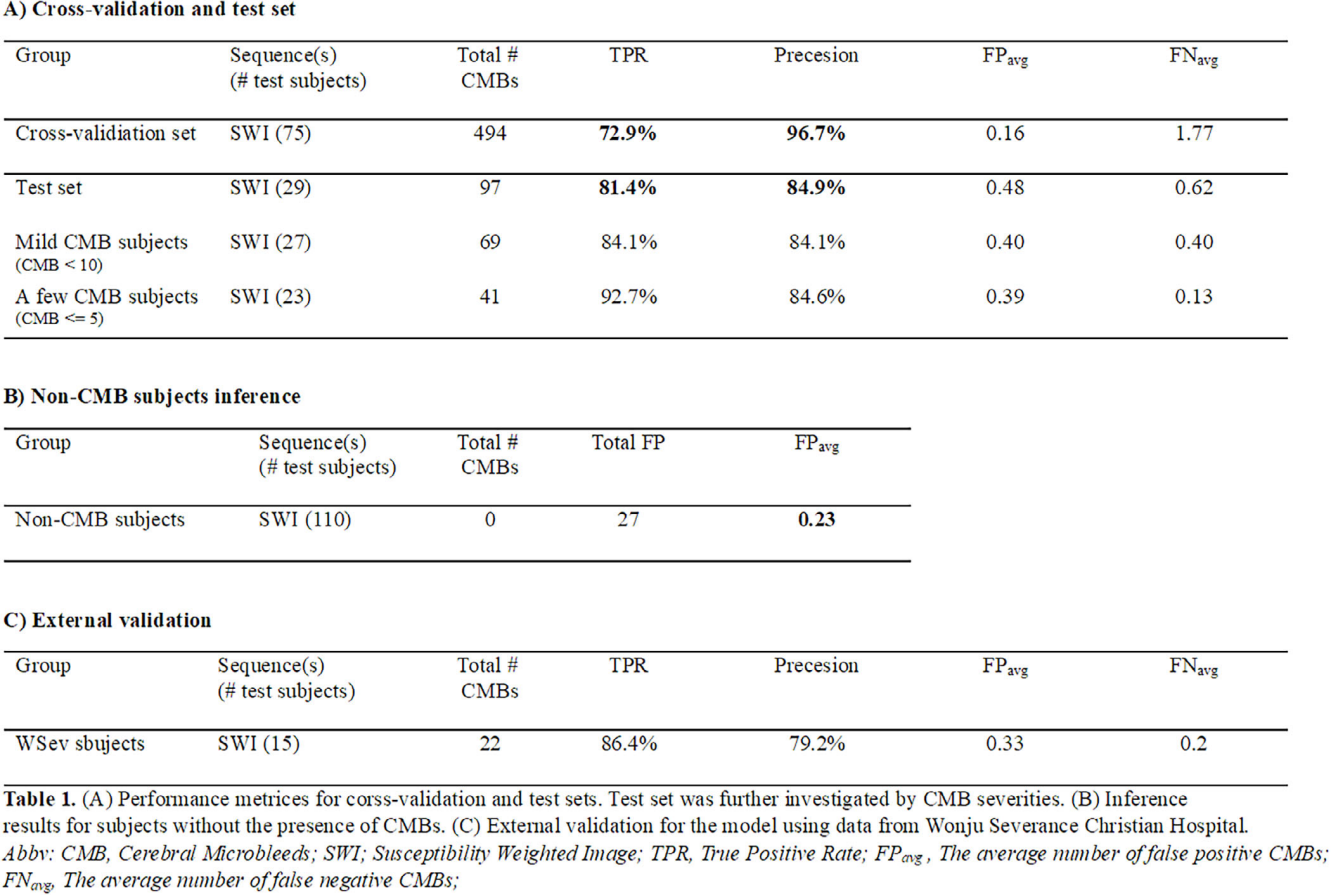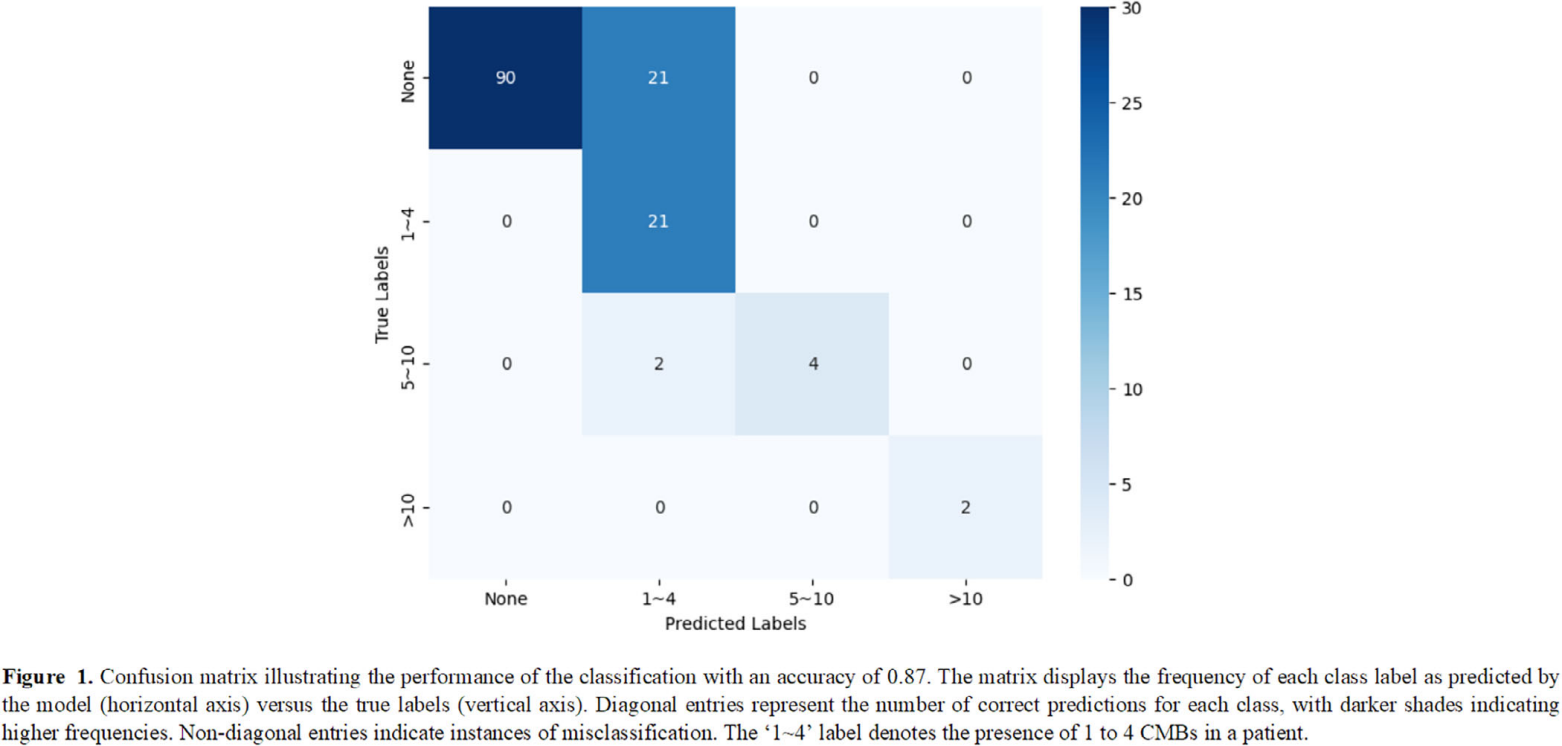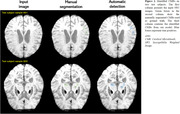# Addressing Unmet Precision Requirements through Improved Automatic Identification of Cerebral Microbleeds

**DOI:** 10.1002/alz.088695

**Published:** 2025-01-09

**Authors:** Younghoon Jeon, Roh‐Eul Yoo, Min Seok Baek, Joon‐Kyung Seong, Seung Hong Choi, Wha Jin Lee

**Affiliations:** ^1^ NeuroXT, Seoul Korea, Republic of (South); ^2^ Seoul National University College of Medicine, Seoul Korea, Republic of (South); ^3^ Seoul National University Hospital, Seoul Korea, Republic of (South); ^4^ Wonju Severance Christian Hospital, Yonsei University Wonju College of Medicine, Wonju Korea, Republic of (South); ^5^ Korea University, Seoul Korea, Republic of (South)

## Abstract

**Background:**

Cerebral microbleeds (CMBs) hold significant clinical relevance, linked to elevated risks of hemorrhages, cognitive decline, and mortality. Notably, with the recent advancement in Alzheimer’s treatments, the number of CMBs serves as a crucial safety indicators to assess the risk and occurrence of amyloid‐related imaging abnormalities. However, the commonly utilized manual detection process is time‐consuming and labor‐intensive, prompting the development of various automated detection models. But, most of them are composed of a two‐track system for candidate retrieval and false positive (FP) reduction, resulting in low precision.

**Method:**

Susceptibility‐weighted images (SWI) were obtained from 250 participants enrolled in the Seoul National University dementia cohort, with 200 and 50 images allocated to the training and test set, respectively. CMBs were segmented manually based on expert radiologist assessments. Only 75 images in the training set and 29 images in the test set revealed the presence of CMBs. For our analysis, we employed 3D‐nnUNet, an segmantic segmentation model, training it through 5‐fold cross validation. The segmented regions were analyzed for CMB counting, focusing on morphological features. External validation was performed using 15 images from Wonju Severance Christian Hospital.

**Result:**

Our model exhibited a concurrent high true positive rate (TPR) of 81.4% and precision of 84.9%. This performance remained consistent across different severities of CMB occurrences, with the best perfomance observed in subjects with five or fewer CMBs (TPR of 92.7% and precision of 84.6%). Additionally, even among non‐CMB subjects, the model demonstrated strong perfomance, yielding an average of 0.23 FPs. External validation confirmed model robustness with a TPR of 86.4% and precision of 79.2% (Table 1). Classification of the severity groups regarding the number of CMBs (None, 1∼4, 5∼10, and >10) revealed an overall accuracy of 87% in the non‐CMB and the test set (Figure 1).

**Conclusion:**

This study introduces a novel single‐channel automated CMB detection model using SWI. Our model successfully tackles a notable challenge found in previous automatic CMB counting models, balancing a high TPR with more reliable precision. It underscores its applicability for accurately stratifying CMB severities in clinical contexts, including eligibility assessment for anti‐beta amyloid antibody treatment in AD.